# Impact of *Elateriospermum tapos* Supplementation on Leptin and Hypothalamic Signaling in Female Offspring of High-Fat Diet-Induced Obese

**DOI:** 10.34172/apb.43919

**Published:** 2025-04-04

**Authors:** Santhra Segaran Balan, Hasnah Bahari, Azrina Zainal Abidin, Nurul Husna Shafie, Maizaton Atmadini Abdullah, Azmiza Syawani Jasni

**Affiliations:** ^1^Department of Diagnostic and Allied Health Science, Faculty of Health and Life Sciences, Management and Science University, Selangor, Malaysia.; ^2^Department of Human Anatomy, Faculty Medicine and Health Sciences, Universiti Putra Malaysia, Selangor, Malaysia.; ^3^Department of Nutrition and Dietetics, Faculty of Medicine and Health Sciences, Universiti Putra Malaysia, Selangor, Malaysia.; ^4^Department of Pathology, Faculty Medicine and Health Sciences, Universiti Putra Malaysia, Selangor, Malaysia.; ^5^Department of Medical Microbiology and Parasitology, Faculty of Medicine and Health Sciences, Universiti Putra Malaysia, Selangor, Malaysia.

**Keywords:** *Elateriospermum tapos*, Maternal obesity, Hypothalamus, Leptin, Obesity

## Abstract

**Purpose:**

The central nervous system plays a crucial role in regulating food intake and energy expenditure to maintain energy homeostasis in the body. With rising obesity rates, alternative therapeutic strategies, including herbal-based interventions, are gaining attention. *Elateriospermum tapos* a plant that rich in flavonoids, has shown potential supporting weight reduction. This study aimed to evaluate the effects of *E. tapos* seed and shell supplementation on the hypothalamic feeding pathway in obese female rats and their offspring.

**Methods:**

Thirty adult female Sprague-Dawley rats were used in this study. Obesity was induced in 24 rats via high-fat diet (HFD) for five weeks. Six rats were maintained on a normal diet as the control group (DCG). The obese rats were then divided into four groups: negative control (DNG, HFD only), positive control (DPG, HFD+orlistat 200 mg/kg), treatment 1 (DTX1, HFD+*E. tapos* seed 200 mg/kg), and treatment 2 (DTX2, HFD+*E. tapos* shell 200 mg/kg). Treatments were administered daily for six weeks before mating. On postnatal day 21 (PND21), blood and hypothalamus samples were collected from female rats and their female offspring. Plasma leptin levels were measured using ELISA, and expression of leptin receptor (Obr), proopiomelanocortin (POMC), and neuropeptide Y (NPY) in the hypothalamus was assessed by western blotting.

**Results:**

DTX2 and offspring (OTX2) groups showed significantly (*P*<0.05) lower levels of leptin. Western blot results indicate Obr, POMC and NPY protein significantly (*P*<0.05) higher expression in DNG and ONG compared to the other groups.

**Conclusion:**

In conclusion, the *E. tapos* shell significantly reduced maternal obesity in female offspring at PND21 compared to its seed.

## Introduction

 Obesity, which is characterized by excessive body fat accumulation, is a major health challenge in contemporary society. This condition is linked to severe health complications including type II diabetes and chronic coronary heart disease. The global obesity epidemic affects both industrialized and developing countries, largely due to modern lifestyle factors, such as physical inactivity and unhealthy eating patterns. Beyond its physical health implications, obesity also carries significant psychological and social ramifications, often diminishing the quality of life and increasing healthcare expenses for individuals and communities. Although poor diet and lack of exercise are commonly cited causes of obesity in adults and adolescents, research suggests that an atypical metabolic environment during fetal development may contribute to its early onset and progression.^1^ Obesity can negatively affect various health markers, including lipid profiles, blood pressure, and metabolic functions, particularly glucose metabolism. It is strongly associated with cardiovascular diseases and type 2 diabetes mellitus. The obesity crisis has extended to children, with worldwide rates of childhood obesity on the rise, potentially setting the stage for lifelong health issues. Moreover, social stigma surrounding obesity can lead to discrimination in various aspects of life, including education and employment. Tackling this complex issue requires a holistic strategy that incorporates public health initiatives, educational programs, and policy changes aimed at promoting healthier lifestyles and environments.^2^ This comprehensive approach should also consider genetic predisposition and environmental influences on weight gain. Furthermore, interventions should emphasize body positivity and mental health support for those struggling with obesity given the substantial psychological impact of weight stigma. Strategies should be tailored to specific age groups and cultural contexts to maximize the efficacy of obesity prevention and management.

 Maternal obesity, defined as excessive weight during pregnancy, presents a significant risk to women of childbearing age. This condition affects both the mothers and their children. Obesogenic transmission, a form of maternal inheritance, can result in obesity in offspring.^3^ Obesity-related characteristics can be passed down through generations. In recent times, childhood obesity has reached epidemic levels owing to its increasing prevalence. Identifying the potential risk factors for obesity is essential for implementing effective preventive measures. Obesity negatively impacts not only children’s physical well-being, but also their social and emotional growth. Children affected by obesity often struggle with low self-esteem and lack confidence in their ability to perform certain tasks.^4^

 Adipocytes primarily produce leptin, a hormone essential for maintaining energy equilibrium. It accomplishes this by conveying feelings of fullness to the hypothalamus, which, in turn, reduces appetite and enhances energy use. When leptin binds to hypothalamic receptors, it triggers various signaling cascades, particularly the JAK/STAT pathway. This pathway influences the expression of neuropeptides that control hunger and satiety. However, obesity often leads to leptin resistance, a condition marked by inadequate leptin signaling despite high circulating leptin levels. This resistance is linked to several factors, including chronic overstimulation of leptin receptors, deficiencies in intracellular signaling, increased inflammation in the hypothalamus, and heightened expression of SOCS3. These elements collectively disrupt the energy balance and contribute to further weight gain.^5^

 Pregnancy-related obesity can change hypothalamic appetite gene expression in the fetus, which is linked to an increased predilection for high-fat, high-sugar junk food.^6^ An early investigation (in vitro) revealed that pancreatic lipase, -glucosidase, and -amylase enzymes were all inhibited by *Elateriospermum tapos*. Oleic acid, -linolenic acid (16.10%), and omega-3 polyunsaturated fatty acids are present in *E. tapos*.^7^ A second study carried out by researchers demonstrated that *E. tapos*, which has a high flavonoid content, aids in fat burning and helps to reduce obesity.^8^ In this study, we investigated the effect of *E. tapos* on protein expression in the hypothalamus, particularly in POMC, NPY, and Obr, in dams and female offspring. First, we examined the plasma levels of leptin in dams and offspring which are directly involved in adipocyte cells. Additionally, we examined protein expression in the brain by western blotting for POMC, NPY, and Obr.

## Methods

###  Identification of plant

 The *E. tapos* plant was obtained from a local farm in Maran Pahang, and the sample was submitted to the Herbarium Biodiversity Unit (UBD, Universiti Putra Malaysia) for identification purposes. The voucher no of this specimen was obtained from our institution (UPM SK 3154/17).

###  Elateriospermum tapos seed and shell extraction using the hot aqueous method.

 The shells and seeds of *E. tapos* were dried before being well-grounded. 50 g of *E. tapos* seeds and shells were mixed with 500 mL of distilled water in a conical flask. This mixture was diluted well and transferred to a 1 L Scott bottle before being placed inside a water bath. The sample was placed in a water bath for 24 h at 70 °C. After 24 hours, the sample was filtered three times using Whatman paper N° 1. Filter sample transfer in Falcon tubes and freeze-drying. The powder was stored at -20 °C in a freezer.^9,10^

###  Maternal group and diet

 Female Sprague Dawley rats were purchased from Saintifik Enterprise (Malaysia) and housed at constant temperature and humidity on a control 12-hour light/dark cycle with free access to food and drink. Female rats were randomly assigned control group to the obese group with a cafeteria diet (CFD) and a high-fat diet (HFD) [N = 24, 1266 kcal/100 g CFD, 414 kcal/100 g] to induce obesity before mating. The control group (DCG) was fed normal chow (N = 6, 306.2 kcal/100 g). After 5 weeks of induction, obese dams were randomly assigned to the four group which is negative control group (DNG), positive control group (DPG) with 200 mg/kg orlistat drug, treatment 1 group (DTX1) with 200 mg/kg *E. tapos*seed, and treatment 2 group (DTX2) with 200 mg/kg *E. tapos*shell for 6 weeks prior to pregnancy. At 15 weeks of age, the females from all groups were mated and maintained on the same diet during pregnancy and lactation.

###  Offspring studies

 Five different groups of offspring were studied in dams fed with different diets and treatments. Offspring from control group (OCG), offspring from negative group (ONG), offspring from positive group (OPG), offspring from treatment 1 group (OTX1) and offspring from treatment 2 (OTX2). For offspring studies, newborns at postnatal day 21 of age (PND 21) (N = 6 liters per group) were used. From each group, all six female offspring were euthanized by rapid decapitation, the brains were removed, and the hypothalamus was dissected and used for analysis. Dams were euthanized on the same day, the brain was removed, and the hypothalamus was dissected for further analysis for comparison with the offspring. Hypothalamus from offspring and dams were snap-frozen and stored at −80 °C for protein expression analysis.

###  ELISA method

 Through cardiac puncture in dams and offspring, 5 ml blood was obtained and placed in heparin tubes. The heparin tube was centrifuged at 2500 RPM for 15 min to obtain plasma for further analysis. Plasma leptin concentration was measured using commercially available ELISA kits (Sigma-Aldrich, St. Louis, MO, USA, RAB0335) according to the manufacturer’s instructions.

###  Western blot 

 The hypothalamus was homogenized in ice-cold RIPA buffer (Tris- HCl, 50 mM; NaCl, 150 mM; EDTA, and Triton X-100, 1%; pH 7.5) containing proteinase inhibitors (Complete, EDTA-free; Roche Applied Science, Indianapolis, IN, USA). The homogenates were subjected to SDS-PAGE, transferred to a nitrocellulose membrane, blocked with 3% BSA in PBS, and reacted with anti-MDA (NOF Corporation), 4-hydroxy-2-hexenal (4-HHE) (NOF Corporation), and anti-β-actin (No. A2228-Sigma-Aldrich) antibodies.^11^ The volume of protein separation was determined to guarantee 50μg of protein in each well. Five microliters of buffer were added to the sample, and the volume in each path was levelled using twofold refined H_2_O (dd H_2_O) and mixed well. The samples were warmed on a dry plate for 5 min at 95 °C. Ten μL of the protein standard was included in the end well of the gel.

 Nitrocellulose membranes with 0.2 µm pore were purchased from Bio-Rad Laboratories, USA. Biotin-labelled goat anti-mouse antibodies (No. SAB4600004-Sigma-Aldrich) and streptavidin alkaline phosphatase were purchased from Sigma-Aldrich. The leptin receptor antibody (No. GTX37636) was purchased from GeneTex. NPY (No. 11976) and POMC (No. 23499) receptor antibodies were purchased from Cell Signaling Technology. The membrane was blocked with freshly prepared 5% skim milk in TBS for 1 hour at 4 °C with constant shaking. The primary antibody was added (Leptin, POMC, or NPY) in 5% skim milk in TBS and was incubated overnight at 4 °C on a shaker. After overnight, the membrane was washed with 0.2% (v/v) TBS at 5 minutes interval three times and removed excess TBS during the third time washing. The later membrane was added with biotin-labeled goat anti-mouse IgG (1:2,500 dilution) in TBS containing 2.5% (w/v) skimmed milk for one hour at room temperature with constant shaking using a shaker. The membrane was washed with 0.2% (v/v) TBS 3 times with 5 min intervals. The protein bands were exposed at the final stage by the KPL 1-component TMB membrane Peroxidase substrate (purchased from sera care). The membrane was immersed using 1 ml substrate per membrane. The bands were visualized using a standard digital camera.

###  Statistics

 Statistical analyses were performed using SPSS 25.0 Windows software (SPSS), and results were expressed as mean ± SEM. All data were analyzed for normality. Protein expression and serum leptin were analyzed by one-way ANOVA, followed by post-hoc least significant difference (LSD) analysis. In all analyses, the probability of *P* < 0.05 was considered statistically significant.

## Results

###  Effect of E. tapos on the plasma leptin concentration in adult dams and offspring at PND21

 As expected, the dams from the DNG group showed a significant increase (*P* < 0.05) in leptin level compared to the DCG group, which correlated with previously reported in same study.^12^ DPG, DTX1, and DTX2 treated rats showed a significant (*P* < 0.05) reduction in plasma leptin concentration compared to DNG rats (Table 1).

**Table 1:** Effects of *E. tapos* treatment on leptin concentration

	**DCG (n=6)**	**DNG (n=6)**	**DPG (n=6)**	**DTX1 (n=6)**	**DTX2 (n=6)**
Leptin (nmol/L)	473.5 ± 12.19 ^a,c^	2159.2 ± 53.16 ^b,c^	953.8 ± 35.00 ^a,b^	949.4 ± 5.73 ^a,b^	953.8 ± 51.57 ^a,b^

Abbreviations: DCG, dams control group; DNG, dams negative control; DPG, dams positive (treatment with Orlistat drug) group; DTX1, dams treatment with seed group; DTX2, dams treatment with shell group. Note: ‘n’ indicated the number of rats for each group. Data are expressed as mean ± SEM and were analyzed by one-way ANOVA, followed by post-hoc LSD. Significant level set at *P* < 0.05. Different superscript letters denote significant differences at *P* < 0.05.

 Plasma leptin concentrations for female offspring were measured at PND21. Female offspring from the ONG group showed a significant increase (*P* < 0.05) compared to those from the OCG group, which correlated with increased body weight. Female offspring from OPG, OTX1, and OTX2 groups had significantly reduced plasma leptin concentrations compared to ONG rats (Table 2). This data showed a similar pattern to the leptin levels in dams.

**Table 2:** Effects of *E. tapos* treatment on leptin concentration

	**OCG (n=6)**	**ONG (n=6)**	**OPG (n=6)**	**OTX1 (n=6)**	**OTX2 (n=6)**
Leptin (nmol/L)	233.6 ± 25.72 ^a,c^	2049.4 ± 374.01 ^b,c^	882.8 ± 30.40 ^a,b^	983.4 ± 125.08 ^a,b^	872.8 ± 28.37 ^a,b^

Abbreviations: OCG, offspring control group; ONG, offspring negative control; OPG, offspring positive (treatment with Orlistat drug) group; OTX1, offspring treatment with seed group; OTX2, offspring treatment with shell group. Note: ‘n’ indicated the number of rats for each group. Data are expressed as mean ± SEM and were analyzed by one-way ANOVA, followed by post-hoc LSD. Significant level set at *P* < 0.05. Different superscript letters denote significant differences at *P* < 0.05.

###  Effect of E. tapos on protein expression of leptin in dams and female offspring

**Figure 1:**
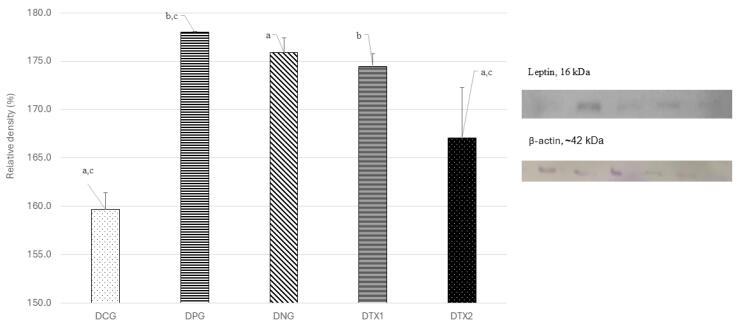
Effect of *E. tapos*supplement on the leptin expression in hypothalamus region of dams. Abbreviations: Letter (D) indicates offspring. CG: Normal Diet; NC: Negative Control; PC: Positive Control; TX1: Treatment 1 (*E. tapos* seed) and TX2: Treatment 2 (*E. tapos* shell). Data are expressed as mean ± SEM and were analyzed by one-way ANOVA, followed by post-hoc LSD. Significant level set at *P* < 0.05. Different superscript letters denote significant differences at *P* < 0.05

 As expected, the optimum expression of the leptin protein band was observed in the DNG group at ~16 kDa, which was close to the expected size of the leptin protein (14 kDa). However, DCG, DPG, DTX1, and DTX2 showed low leptin protein band expression. This can be seen in Figure 1, with the band observed at ~16 kDa along with a picture of the β-actin band. Quantitative assessment was performed on the blots using Image J software to measure the relative density of leptin protein expression. Leptin protein expression was significantly higher (*P* < 0.05) in the DNG group than that in the other groups. This shows that leptin expression was higher in obese rats compared to DCG and DPG, DTX1 and DTX2 treatment groups (Figure 1).

**Figure 2:**
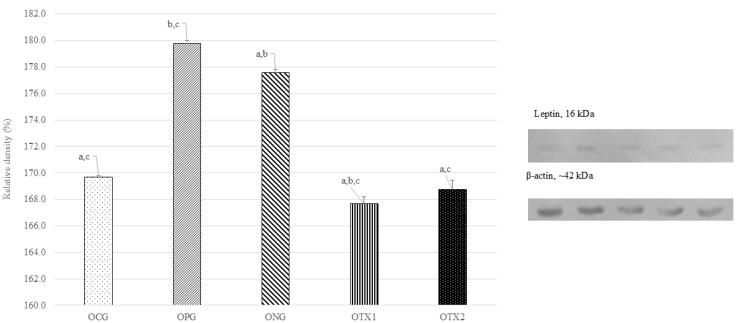
Effect of *E. tapos* supplement on the Leptin expression in hypothalamus region of female offspring at PND21. Abbreviations: Letter (O) indicates offspring. CG: Normal Diet; NC: Negative Control; PC: Positive Control; TX1: Treatment 1 (*E. tapos* seed) and TX2: Treatment 2 (*E. tapos* shell). Data are expressed as mean ± SEM and were analyzed by one-way ANOVA, followed by post-hoc LSD. Significant level set at *P* < 0.05. Different superscript letters denote significant differences at *P* < 0.05

 A similar investigation was conducted using a female offspring brain sample. As expected, the optimum expression of the leptin protein band was observed in the ONG group at ~16 kDa, which corresponds to the expected size of the leptin protein (14 kDa). However, OCG, OPG, OTX1, and OTX2 showed low levels of leptin protein band expression. This result was consistent with result from dams group. The relative density of leptin protein expression was significantly higher (*P* < 0.05) in the ONG group than that in the other groups. This shows that leptin was expressed at higher levels in obese rats than in the standard and treatment groups (Figure 2).

###  Effect of E. tapos on protein expression of POMC in dams and female offspring

**Figure 3:**
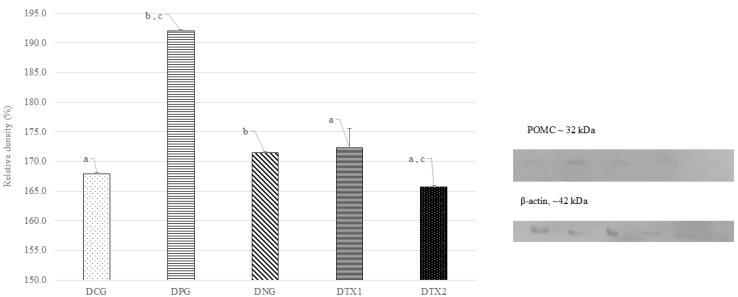
Effect of *E. tapos* supplement on the POMC expression in hypothalamus region of dams. Abbreviations: Letter (D) indicates dams. CG: Normal Diet; NC: Negative Control; PC: Positive Control; TX1: Treatment 1 (*E. tapos* seed) and TX2: Treatment 2 (*E. tapos* shell). Data are expressed as mean ± SEM and were analyzed by one-way ANOVA, followed by post-hoc LSD. Significant level set at *P* < 0.05. Different superscript letters denote significant differences at *P* < 0.05

 The POMC protein band showed optimal expression in the DNG group at approximately 32 kDa, which is consistent with what is anticipated for the POMC protein in terms of size (32 kDa). The POMC protein band was only weakly expressed in DCG, DPG, DTX1, and DTX2 rats. Figure 3 illustrates this with a photograph of the SDS-PAGE gel, with a band visible at approximately 32 kDa. Dams in the DNG group exhibited considerably higher (*P* < 0.05) POMC protein expression than those in the other groups. This demonstrated that obese dams expressed more POMC than normal or treated animals (Figure 3).

**Figure 4:**
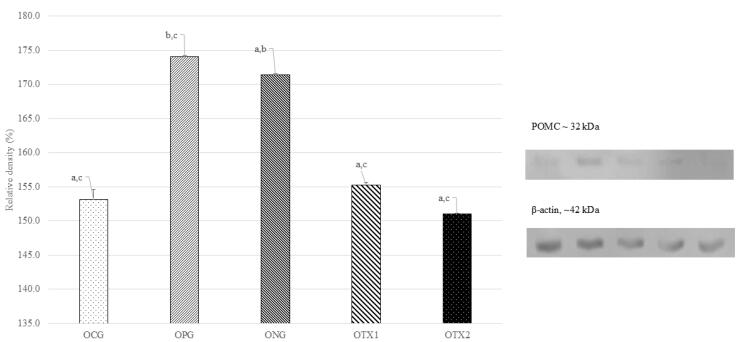
Effect of *E. tapos* supplement on the POMC expression in hypothalamus region of female offspring at PND21. Abbreviations: Letter (O) indicates offspring. CG: Normal Diet; NC: Negative Control; PC: Positive Control; TX1: Treatment 1 (*E. tapos* seed) and TX2: Treatment 2 (*E. tapos* shell). Data are expressed as mean ± SEM and were analyzed by one-way ANOVA, followed by post-hoc LSD. Significant level set at *P* < 0.05. Different superscript letters denote significant differences at *P* < 0.05

 For offspring, the ONG group showed the expression of the POMC protein band at approximately 32 kDa, which is consistent with the predicted size of the POMC protein (32 kDa). The POMC protein band were weakly expressed in OCG, OPG, OTX1, and OTX2 rats. Figure 4 illustrates this with a photograph of the SDS-PAGE gel, with a band visible at approximately 32 kDa. Offspring in the DNG group exhibited considerably higher (*P* < 0.05) POMC protein expression than offspring in the other groups. This demonstrated that obese offspring expressed more POMC than normal or treated offspring (Figure 4).

###  Effect of E. tapos on protein expression of NPY in dams and female offspring

**Figure 5:**
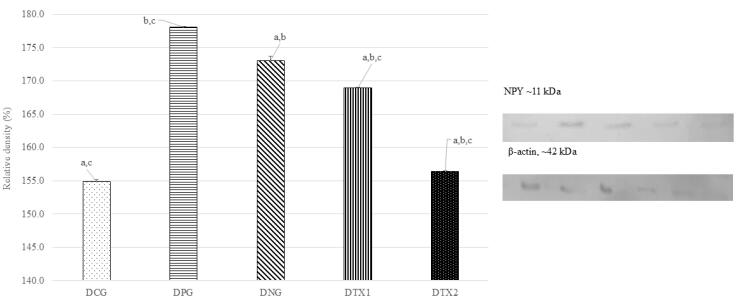
Effect of *E. tapos*supplement on the NPY expression in hypothalamus region of dams. Abbreviations: Letter (D) indicates dams. CG: Normal Diet; NC: Negative Control; PC: Positive Control; TX1: Treatment 1 (*E. tapos* seed) and TX2: Treatment 2 (*E. tapos* shell). Data are expressed as mean ± SEM and were analyzed by one-way ANOVA, followed by post-hoc LSD. Significant level set at *P* < 0.05. Different superscript letters denote significant differences at *P* < 0.05

 The DNG group showed the highest level of NPY protein expression, which matched the predicted size of around 11 kDa (11 kDa). The NPY protein band was expressed at modest levels in DCG, DPG, DTX1, and DTX2. Figure 5 illustrates this with a photograph of the SDS-PAGE gel, with a band visible at approximately 11 kDa. Dams in the DNG group exhibited considerably higher NPY protein expression than those in the other groups (*P* < 0.05). This demonstrated that obese dams expressed more NPY than normal or treated animals (Figure 5).

**Figure 6:**
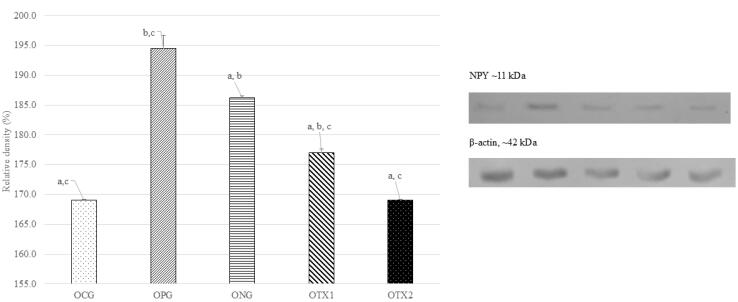
Effect of *E. tapos* supplement on the NPY expression in hypothalamus region of female offspring at PND21. Abbreviations: Letter (O) indicates offspring. CG: Normal Diet; NC: Negative Control; PC: Positive Control; TX1: Treatment 1 (*E. tapos*seed) and TX2: Treatment 2 (*E. tapos* shell). Data are expressed as mean ± SEM and were analyzed by one-way ANOVA, followed by post-hoc LSD. Significant level set at *P* < 0.05. Different superscript letters denote significant differences at *P* < 0.05

 At 11 kDa, the predicted size of the NPY protein, the ONG group showed the highest expression of the NPY protein band (11 kDa). The NPY protein band was expressed at low levels in the OCG, OPG, OTX1, and OTX2. Figure 6 illustrates this with a photograph of the SDS-PAGE gel, with a band visible at approximately 11 kDa. Offspring in the ONG group showed considerably higher (*P* < 0.05) levels of NPY protein expression than those in the other groups. This demonstrated that obese offspring expressed more NPY than normal or treated animals (Figure 6).

## Discussion

 Adiposity accumulation and excessive dietary fat consumption are two characteristics of the complex condition known as obesity in humans.^13^ Chronic inflammation and adipocyte hypoxia, which result in dysregulation of adipokine synthesis and activation of the pro-inflammatory pathway, are additional conditions associated with obesity.^14^ Obesity is typically linked to an increased risk of mortality and morbidity from diabetes mellitus and cardiovascular diseases, such as hypertension, stroke, coronary heart disease, and numerous malignancies.^15^ As obesity is the source of many ailments, its management is essential. The overall adverse effects of conventional treatment for obesity, including headache, constipation, cardiac arrhythmia, and other complications, have been associated with the increased utilization of traditional herbal therapy.^16^ Consequently, natural remedies that are relatively safe and have fewer side effects are becoming increasingly prevalent to address obesity. There were two primary areas of focus for this investigation. In this study, we examined the effect of *E. tapos*supplementation in dams prior to pregnancy on their feeding circuitry and the subsequent effects on next-generation offspring.

 The hypothalamus is a crucial component of the feeding circuitry responsible for regulating energy intake and expenditure in both rats and humans. Both orexigenic neuropeptide Y (NPY)/agouti-related peptide (AgRP), anorexigenic proopiomelanocortin (POMC)/cocaine, and amphetamine-regulated transcript (CART) neuropeptides are produced in the hypothalamus by two interconnected neurons. NPY directly contributes to the upregulation of food intake in both humans and animals by enhancing energy intake, which involves the Y1 and Y5 receptors.

 In the presence of insulin, the adipocyte hormone leptin inhibits AMPK in the hypothalamus, thereby preventing overeating and reducing appetite. The expression of leptin affects its ability to induce satiety and prevent obesity.^17^ In infants with HFD-induced obese mothers, elevated leptin hormone levels during breastfeeding induced rapid weight gain, according to a study by Lecoutre et al.^18^ Because it helps the offspring of obese dams reduce their food consumption, regulation of leptin and AMPK activity is important. According to the current study, obese dams and their female offspring had higher levels of leptin hormone expression than the control and treatment groups. Previous study done by Balan et al,^10^ shows the increasing in body weight of dams is correlated with increasing the expression of leptin. The researcher also concludes that body weight, total adipocyte and leptin have correlated to each other. In dams and female offspring, treatment with *E. tapos*significantly lowered the body weight and leptin expression. The results of the current study demonstrated that supplementation with *E. tapos*, which decreased body weight and adiposity, decreased leptin levels in dams and female offspring.

 Increased parasympathetic activity, hunger, and food and energy intake are all effects of increased NPY expression in the hypothalamus, but sympathetic activity and energy expenditure are suppressed. As a result, energy storage increases. Leptin is an adipocyte-derived polypeptide that inhibits this function. Leptin receptor resistance also results from increased levels of leptin. Increased hunger and energy consumption result from leptin receptor resistance. In both humans and animals, POMC is essential for food intake and energy expenditure. The POMC neuron, which collaborates with leptin, is situated in the arcuate nucleus of the hypothalamus. Together with leptin, POMC is expressed in the arcuate nucleus. Therefore, POMC is primarily involved in satiety. Study conducted by Gallo et al^19^ showed that POMC neurons produce IL-17A, and the decreased number of POMC neurons in POMC-Cre mice may be associated with reduced IL-17A production. This reduction in IL-17A corresponds to a decrease in adipocyte gene expression associated with obesity.

 According to recent research, HFD or cafeteria food consumption might cause an inflammatory response in the hypothalamus, which disrupts the thermogenic and anorexigenic signals produced by these hormones, resulting in aberrant body weight. This study also demonstrated that treatment with *E. tapos* shells or seeds decreased the elevated brain expression of an inflammatory marker in offspring, which is linked to maternal obesity. Compared with the other treatment groups in these trials, the DPG and OPG groups displayed the highest levels of leptin protein and NPY expression. This demonstrates how well *E. tapos* seed and shell suppressed adipocyte and NPY expression in the hypothalamus of dams and female offspring.^12^ Tiesjema et al^20^ in his study also mention that when NPY signaling is enhanced in the PVN of rats through rAAV-mediated NPY overexpression, it leads to excessive eating and weight gain in rats. This process involves the use of recombinant adeno-associated viruses to increase neuropeptide Y levels in the paraventricular nucleus, resulting in hyperphagia and obesity in the affected rats.

 In the current study, the significant impact of *E. tapos* was examined as a potential anti-obesity agent to reduce maternal obesity in female rat offspring PND21 by preventing adipogenesis. This research shows that *E. tapos* aids in reducing inflammation in the hypothalamus because consumption of HFD or cafeteria food alters the changes in the hypothalamus. However, in this study we have limitations which only focus hypothalamic inflammation compared to other inflammation markers. To enhance this, further study of other inflammation markers may be conducted.

## Competing Interests

 No potential conflict of interest relevant to this article was reported.

## Ethical Approval

 This study was approved by the Institutional Animal Care and Use Committee at the Management & Science University (MSU) (Animal Ethics No. AE-MSU-073). The study was conducted in accordance with proper animal care guidelines.
